# Preparation of the Valerianate of Iron

**Published:** 1846-12

**Authors:** 


*Preparation of the Valerianate of Iron, by M. Ruspini.*—In the *Memoriale della Medicini Contemporanea,* M. Ruspini gives the following prescription for the preparation of the valerianate of iron:—Put four grammes of iron filings into a small porcelain mortar, and pour on them by degrees an equal quantity of valerianic acid, at the same time continually stirring. In about a quarter of an hour the mixture presents the tenacity of glue. On the addition of the first drops of the acid a powerful odour of acetic acid is given off, which becomes still stronger in proportion as a greater quantity of acid is added. At the expiration of an hour, during which time it is necessary to continue stirring, the product acquires a dull red colour. Distilled water must then be added to the mixture, in order better to incorporate the solid matter which adheres to the sides of the vessel. The mixture must then be put into a proper vessel, heated, and filtered soon after. The filtered fluid, which contains the valerianate of the protoxide of iron, passes clear, slightly acid, and of a styptic, but by no means disagreeable, taste. On cooling, whilst exposed to the air, it becomes covered by degrees with a crystalline layer of a red colour. This is the first portion of the valerianate of peroxide of iron, which begins to separate itself from the fluid, being produced by the too active oxidation of the salt of iron. The fluid must be a second time filtered to separate this. The liquid remaining becomes again covered with a pellicle, which must be again removed, and so on. To hasten the process the liquid may be concentrated by heat. The valerianate of iron obtained by this mode possesses a reddish colour. It is partly in powder, and partly in the form of brilliant scales; it has a styptic taste, and a scarcely sensible smell of valerianic acid, and is insoluble in water. A few grains treated with a drop of concentrated sulphuric acid, give out the strong and characteristic odour of valerianic acid.—*Ibid.*

